# Impact of left ventricular diastolic function and direct oral anticoagulant use for predicting embolic events in patients with heart failure and atrial fibrillation

**DOI:** 10.1002/joa3.13031

**Published:** 2024-03-26

**Authors:** Jumpei Yamamoto, Hiromasa Hayama, Yoshinari Enomoto, Masaya Yamamoto, Hisao Hara, Yukio Hiroi

**Affiliations:** ^1^ Department of Cardiology National Center for Global Health and Medicine Tokyo Japan

**Keywords:** atrial fibrillation, atrial remodeling, echocardiography, heart failure, ischemic stroke

## Abstract

**Background:**

Patients with atrial fibrillation (AF) and heart failure (HF) have high stroke risk owing to left atrial dysfunction. However, anticoagulation is a concern in patients with high bleeding risk. We aimed to identify independent predictors of stroke in HF patients with AF.

**Methods:**

We retrospectively examined 320 patients (mean age 79 ± 12 years, 163 women) hospitalized with acute HF complicated by AF between January 2014 and December 2018. Patients were followed from admission until ischemic stroke or systemic embolism (SSE) onset or death or were censored at the last contact date or September 2023.

**Results:**

SSE occurred in 40 patients (median follow‐up of 528 days). Multivariate Cox regression analysis identified age (hazard ratio [HR] 1.04, 95% confidence interval [CI] 1.00–1.07, *p* = .034), direct oral anticoagulant (DOAC) use (HR 0.26, 95% CI 0.11–0.60, *p* = .002), and early diastolic peak flow velocity to early diastolic peak annular velocity (E/e'; HR 1.05, 95% CI 1.02–1.08, *p* < .001) to be independent predictors of SSE, whereas left atrial reservoir strain was not. After determining an appropriate E/e' cutoff by receiver‐operating characteristic curve analysis and adjusting the multivariate Cox model, E/e' ≥17.5 (HR 3.30, 95% CI 1.56–6.83, *p* = .001) independently predicted SSE. The results were consistent with no interaction in the subanalysis except for gender.

**Conclusion:**

Elderly patients not on DOACs with elevated E/e' may be at higher risk of stroke, suggesting that DOACs should be the first choice for patients with elevated E/e' and aggressive additional prophylaxis and careful follow‐up are needed.

## Graphical abstract

We investigated independent predictors of ischemic stroke or systemic embolism (SSE) in patients hospitalized for acute heart failure complicated by atrial fibrillation. Multivariate analysis showed that age, no DOAC use, and E/e' were significant independent predictors of SSE, and E/e' may be better than left atrial strain for assessment of SSE risk in these patients. Elderly patients with increased E/e' who are not on DOACs may be at higher risk of SSE and need aggressive treatment and careful follow‐up.

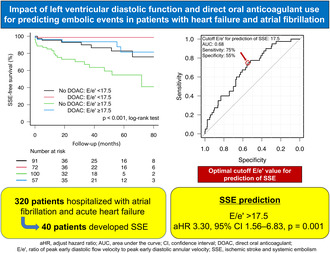

## 1 INTRODUCTION

Atrial fibrillation (AF) increases the risk of cardiogenic stroke fivefold.^1^ Anticoagulation is highly effective in preventing stroke in patients with AF, with risk stratification based on the CHADS_2_ score determining the indication.^2^ However, given that stroke has been reported even in patients with a CHADS_2_ score of 0,^3^ other risk factors should also be evaluated when considering the indication for oral anticoagulants (OACs).^2^ It has also been reported that patients on anticoagulation therapy have an annual stroke rate of about 2%,^4^ suggesting the usefulness of early rhythm control and left atrial (LA) appendage closure as additional prevention measures.^5^, ^6^ The use of the existing scoring system alone is not sufficient for early detection of patients at high risk of stroke, so other modalities are needed.^7^

The ratio of early diastolic peak flow velocity to early diastolic peak annular velocity (E/e') on tissue Doppler echocardiography reflects left ventricular (LV) end‐diastolic pressure and is an indicator of LV diastolic dysfunction.^8^ Elevated E/e' is associated with the cardiovascular prognosis^9^ and has been reported to be superior to the CHADS_2_ score as an independent predictor of LA appendage thrombus and stroke in patients with AF.^10^, ^11^, ^12^ We have previously reported that the LA reservoir strain (LARS) obtained from two‐dimensional speckle tracking is better than E/e' for the prediction of cardiovascular events in patients with heart failure (HF) and AF.^13^ Early rhythm control may be desirable in this patient group because of the poor prognosis resulting from LA dysfunction, but it is unclear whether there is a need for routine checking for LA appendage thrombus before AF ablation or electrical cardioversion. Furthermore, there are few reports on whether E/e', LARS, or use of direct OACs (DOACs) is associated with the risk of stroke in this patient population. Therefore, in this study, we aimed to identify prognostic factors for stroke in patients with HF and AF.

## 2 METHODS

### 2.1 Patients

The study had a single‐center, retrospective, observational design, the details of which were described in our previous reports.^13^, ^14^ Briefly, the study included patients aged 18 years or older who were admitted to the Department of Cardiology at the Center Hospital of the National Center for Global Health and Medicine (Tokyo, Japan) with acute HF complicated by AF between January 2014 and December 2018. Exclusion criteria were acute coronary syndrome, no transthoracic echocardiography performed within 30 days before or after admission, and missing data. We extended the follow‐up duration of our previous study to September 2023, either from the time of admission until death or ischemic stroke or systemic embolism (SSE) or until the date of last contact. Follow‐up events were the onset of SSE and pulmonary vein isolation by catheter ablation.

The study was approved by the institutional review board of the National Center for Global Health and Medicine (NCGM‐S‐004777‐00) and conducted in accordance with the Declaration of Helsinki.

### 2.2 Echocardiography measurements

The measurement methods used in transthoracic echocardiography and the reproducibility and reliability of strain measurements were described in detail in our previous report.^13^ Briefly, transthoracic echocardiography was performed by echocardiographers using an Aplio400® or Artida® ultrasound system (Toshiba Medical Systems). LV lumen measurements were obtained by the modified Simpson method. LV weight was calculated by the modified Devereux method. LA diameter was measured from the long‐axis view, and LA volume was measured by the biplane area‐length method. Peak early diastolic annular velocity was measured at the mitral annulus septum. LARS was measured offline from two‐ and four‐chamber views and LV global longitudinal strain (LVGLS) from two‐, three‐, and four‐chamber views using the AutoStrain function in the two‐dimensional speckle tracking software Image Arena® (TOMTEC Imaging Systems, Unterschleissheim). Mean LARS was calculated as an absolute value from two‐ and four‐chamber views and used for analysis. The intra‐observer correlation coefficient confirmed high reproducibility of the strain measurement and high reliability of the heart rate for each image section.

### 2.3 Definitions

Patient characteristics other than medications at discharge were based on data obtained at the time of admission. Acute HF was diagnosed on admission by cardiologists at our hospital based on the Framingham criteria.^15^ The diagnosis of AF was made by 12‐lead electrocardiogram or transthoracic echocardiography on admission. Calculation of time in the therapeutic range for warfarin and other disease definitions have also been reported previously.^13^, ^14^

### 2.4 Statistical analysis

Continuous variables are shown as the mean ± standard deviation or median [interquartile range] and categorical variables as the frequency (percentage). We compared baseline variables between groups according to whether SSE occurred using the Student's *t*‐test or Mann–Whitney *U* test if they were continuous and Fisher's exact test if they were categorical. A multivariate model that included age, gender, and other clinical factors that were either significant in univariate Cox analysis or considered to be important was used to identify independent risk factors for SSE. Hazard ratios (HRs) and 95% confidence intervals (CIs) were calculated. Among the echocardiographic values, independent factors for SSE will be adjusted in the multivariate model of the main analysis by dividing the patients into an event group and a no‐event group using the optimal cutoff value identified by receiver‐operating characteristic (ROC) curve analysis. The risk of SSE was also compared between the event group and the no‐event group according to whether a DOAC was used with adjustment of the multivariate model used for the main analysis. Kaplan–Meier curves showing the incidence of SSE in each group were drawn, and log‐rank tests were performed. To evaluate the interaction, subgroup analysis was performed by dividing the event group and the no‐event group according to categorical variables or clinically meaningful or median values for continuous variables. Sensitivity analysis was performed by including the CHA₂DS₂–VASc score, use of OACs, warfarin, and antiplatelets, LV ejection fraction (LVEF), LA volume index, LARS, and LVGLS in the multivariate models. An additional sensitivity analysis was performed with patients in whom echocardiography was performed after admission. All statistical analyses were performed using R version 4.1.2 (The R Foundation for Statistical Computing). All tests were two‐tailed, and a *p*‐value of <.05 was considered statistically significant.

## 3 RESULTS

### 3.1 Patient characteristics

A total of 320 patients (mean age 79 ± 12 years, 163 women) were included in the analysis. During a median follow‐up of 528 days, 2.5% of patients (8/320) underwent pulmonary vein isolation by catheter ablation and 13% (40/320) developed SSE (cardiogenic stroke, *n* = 33; atherothrombotic stroke, *n* = 3; systemic embolism, *n* = 4). Table 1 shows the patient demographic and clinical characteristics. The median duration of AF was 1.2 years, and 11% (36/320) had paroxysmal AF. Hypertension was present in 74% of patients and diabetes mellitus in 30%, with a history of stroke documented in 14%. The median CHA₂DS₂–VASc score was 5, and 84% of patients were on an OAC. The event group contained significantly greater numbers of women and warfarin users and fewer patients on DOACs. There was no significant difference in age, estimated glomerular filtration rate, hemoglobin, comorbidities, CHA₂DS₂–VASc score, or inappropriate dosing of DOAC between the event and no‐event groups. Table 2 shows the transthoracic echocardiographic parameters. The median time from admission to echocardiography was 6 days (IQR 3–9 days). The mean LVEF was 45% and the mean LA diameter was 49 mm. E/e' was significantly elevated in the event group. There was no significant difference in LVGLS or LARS between the event group and the no‐event group.

**TABLE 1:** Comparison of demographic and clinical characteristics according to whether patients developed SSE events.

Variable	Overall (*n* = 320)	Event group (*n* = 40)	No‐event group (*n* = 280)	*p‐*value
Age, years	79.0 ± 11.7	80.3 ± 9.2	78.8 ± 12.0	.46
Female gender, *n*	163 (50.9)	27 (67.5)	136 (48.6)	**.028**
Weight, kg	52.7 ± 15.6	51.1 ± 12.9	52.9 ± 16.0	.49
Body mass index	21.5 ± 4.9	22.0 ± 4.8	21.4 ± 4.9	.45
Heart rate, bpm	97 ± 30	83 ± 26	99 ± 30	**.001**
Blood pressure, mmHg				
Systolic	129 ± 26	131 ± 35	129 ± 25	.60
Diastolic	82 ± 21	78 ± 20	83 ± 21	.17
NYHA functional class				.79
II, *n*	38 (11.9)	6 (15.0)	32 (11.4)	
III, *n*	117 (36.6)	14 (35.0)	103 (36.8)	
IV, *n*	165 (51.6)	20 (50.0)	145 (51.8)	
Duration of AF, years	1.2 [0.0, 5.4]	2.0 [0.0, 6.0]	1.0 [0.0, 5.1]	.41
Type of AF				.25
Paroxysmal, *n*	36 (11.2)	6 (15.0)	30 (10.7)	
Persistent, *n*	132 (41.2)	12 (30.0)	120 (42.9)	
Permanent, *n*	152 (47.5)	22 (55.0)	130 (46.4)	
Postdischarge rhythm control				
Catheter ablation, *n*	8 (2.5)	0 (0.0)	8 (2.9)	.60
Electrical cardioversion, *n*	14 (4.4)	3 (7.5)	11 (3.9)	.40
Antiarrhythmics, *n*	35 (10.9)	5 (12.5)	30 (10.7)	.79
History of smoking, *n*	136 (42.5)	14 (35.0)	122 (43.6)	.39
eGFR, ml/min/1.73 m^2^	46.4 ± 20.9	46.7 ± 21.4	46.3 ± 20.9	.92
Hemoglobin, mg/dL	12.2 ± 2.3	11.8 ± 2.3	12.3 ± 2.3	.20
BNP, pg/mL	623 [397, 1027]	622 [220, 1042]	623 [406, 1021]	.49
Vascular disease, *n*	76 (23.8)	7 (17.5)	69 (24.6)	.43
Coronary artery disease, *n*	71 (22.2)	7 (17.5)	64 (22.9)	.54
Lower extremity artery disease, *n*	7 (2.2)	0 (0.0)	7 (2.5)	.60
Valvular heart disease, *n*	101 (31.6)	16 (40.0)	85 (30.4)	.28
Hypertrophic cardiomyopathy, *n*	7 (2.2)	2 (5.0)	5 (1.8)	.21
Dilated cardiomyopathy, *n*	6 (1.9)	1 (2.5)	5 (1.8)	.55
Comorbidity				
Hypertension, *n*	236 (73.8)	28 (70.0)	208 (74.3)	.57
Dyslipidemia, *n*	103 (32.2)	13 (32.5)	90 (32.1)	1.00
Diabetes, *n*	96 (30.0)	12 (30.0)	84 (30.0)	1.00
COPD, *n*	24 (7.5)	2 (5.0)	22 (7.9)	.75
History of stroke, *n*	44 (13.8)	7 (17.5)	37 (13.2)	.46
CHADS_2_ score	3 [2, 4]	3 [2, 4]	3 [2, 4]	.80
CHA₂DS₂‐VASc score	5 [4, 6]	5 [4, 6]	5 [4, 6]	.48
HAS‐BLED score	2 [2, 3]	3 (2–3)	2 [2–3)	.28
Medication at discharge				
OAC, *n*	269 (84.1)	31 (77.5)	238 (85.0)	.25
DOAC, *n*	129 (40.3)	7 (17.5)	122 (43.6)	**.002**
Inappropriate dosing, *n*	24 (7.5)	3 (7.5)	21 (7.5)	.12
Contraindication, *n*	2 (0.6)	0 (0.0)	2 (0.7)	1.00
Warfarin, *n*	140 (43.8)	24 (60.0)	116 (41.4)	**.040**
TTR, %	57 ± 27	60 ± 27	56 ± 27	.56
PT‐INR	1.95 (0.31)	1.85 (0.27)	1.98 (0.32)	.069
Antiplatelets, *n*	90 (28.1)	15 (37.5)	75 (26.8)	.19
Diuretics, *n*	293 (91.6)	37 (92.5)	256 (91.4)	1.00
Loop diuretics, *n*	287 (89.7)	37 (92.5)	250 (89.3)	.78
MRA, *n*	146 (45.6)	18 (45.0)	128 (45.7)	1.00
Thiazides, *n*	27 (8.4)	4 (10.0)	23 (8.2)	.76
Tolvaptan, *n*	39 (12.2)	5 (12.5)	34 (12.1)	1.00
Beta‐blockers, *n*	235 (73.4)	28 (70.0)	207 (73.9)	.57
ACEI/ARB, *n*	131 (40.9)	20 (50.0)	111 (39.6))	.23
SGLT2 inhibitors, *n*	6 (1.9)	1 (2.5)	5 (1.8)	.55
Calcium channel blockers, *n*	128 (40.0)	19 (47.5)	109 (38.9)	.31
Statins, *n*	89 (27.8)	15 (37.5)	74 (26.4)	.19

*Note*: Data are presented as the number (percentage), mean ± standard deviation, or median [interquartile range]. Values in bold are statistically significant at *p* < .05.

Abbreviations: ACEI, angiotensin‐converting enzyme inhibitor; AF, atrial fibrillation; ARB, angiotensin receptor blocker; BNP, brain natriuretic peptide; COPD, chronic obstructive pulmonary disease; DOAC, direct oral anticoagulant; eGFR, estimated glomerular filtration rate; MRA, mineralocorticoid receptor antagonist; NYHA, New York Heart Association; OAC, oral anticoagulants; PT‐INR, prothrombin time‐international normalized ratio; SGLT2, sodium–glucose cotransporter 2; SSE, ischemic stroke or systemic embolism; TTR, time in therapeutic range.

**TABLE 2:** Echocardiographic data according to development of SSE.

Echocardiographic parameter	Overall (*n* = 320)	Event group (*n* = 40)	No‐event group (*n* = 280)	*p‐*value
LVDd, mm	49.8 ± 9.5	49.8 ± 8.6	49.8 ± 9.6	.99
LVDs, mm	37.9 ± 10.8	36.2 ± 9.8	38.2 ± 11.0	.28
LVEDV, ml	111.7 ± 57.7	99.4 ± 55.3	113.5 ± 57.9	.15
LVESV, ml	65.8 ± 47.1	55.6 ± 46.1	67.3 ± 47.2	.15
LVEF, %	44.9 ± 13.9	46.7 ± 14.3	44.7 ± 13.8	.38
IVSTd, mm	10.4 ± 2.0	10.7 ± 1.9	10.4 ± 2.0	.29
LVPWTd, mm	10.4 ± 1.6	10.5 ± 1.7	10.4 ± 1.6	.67
LVMI, g/m^2^	130.4 ± 42.5	138.1 ± 46.0	129.3 ± 41.9	.22
LAD, mm	49.2 ± 9.2	51.0 ± 10.2	49.0 ± 9.0	.20
LAVI, ml/m^2^	89.7 ± 56.1	97.2 ± 56.0	88.6 ± 56.2	.37
Mitral E velocity, cm/s	109.1 ± 36.0	126.8 ± 45.9	106.6 ± 33.7	**.001**
DcT, ms	201.7 ± 80.8	242.1 ± 133.4	195.9 ± 68.6	**.001**
Septal e' velocity, cm/s	6.0 ± 1.9	5.3 ± 1.4	6.1 ± 2.0	**.017**
E/e' ratio	19.7 ± 8.8	25.0 ± 11.3	19.0 ± 8.1	**<.001**
LVGLS, %	−11.8 ± 6.5	−12.2 ± 6.8	−11.6 ± 6.4	.54
LARS, %	9.6 ± 4.7	9.6 ± 4.7	9.6 ± 4.7	.96

*Note*: Data are presented as the number (percentage) or as the mean ± standard deviation. Values in bold are statistically significant at *p* < 0.05.

Abbreviations: DcT, deceleration time; E, early diastolic peak flow velocity; e', early diastolic peak annular velocity; IVSTd, interventricular septal thickness at end‐diastole; LAD, left atrial diameter; LARS, left atrial reservoir strain; LAVI, left atrial volume index; LVDd, left ventricular dimension in diastole; LVDs, left ventricular dimension in systole; LVEDV, left ventricular end‐diastolic volume; LVEF, left ventricular ejection fraction; LVESV, left ventricular end‐systolic volume; LVGLS, left ventricular global longitudinal strain; LVMI, left ventricular mass index; LVPWTd, left ventricular posterior wall thickness at end‐diastole; SSE, ischemic stroke or systemic embolism.

### 3.2 Predictors of SSE

**FIGURE 1:**
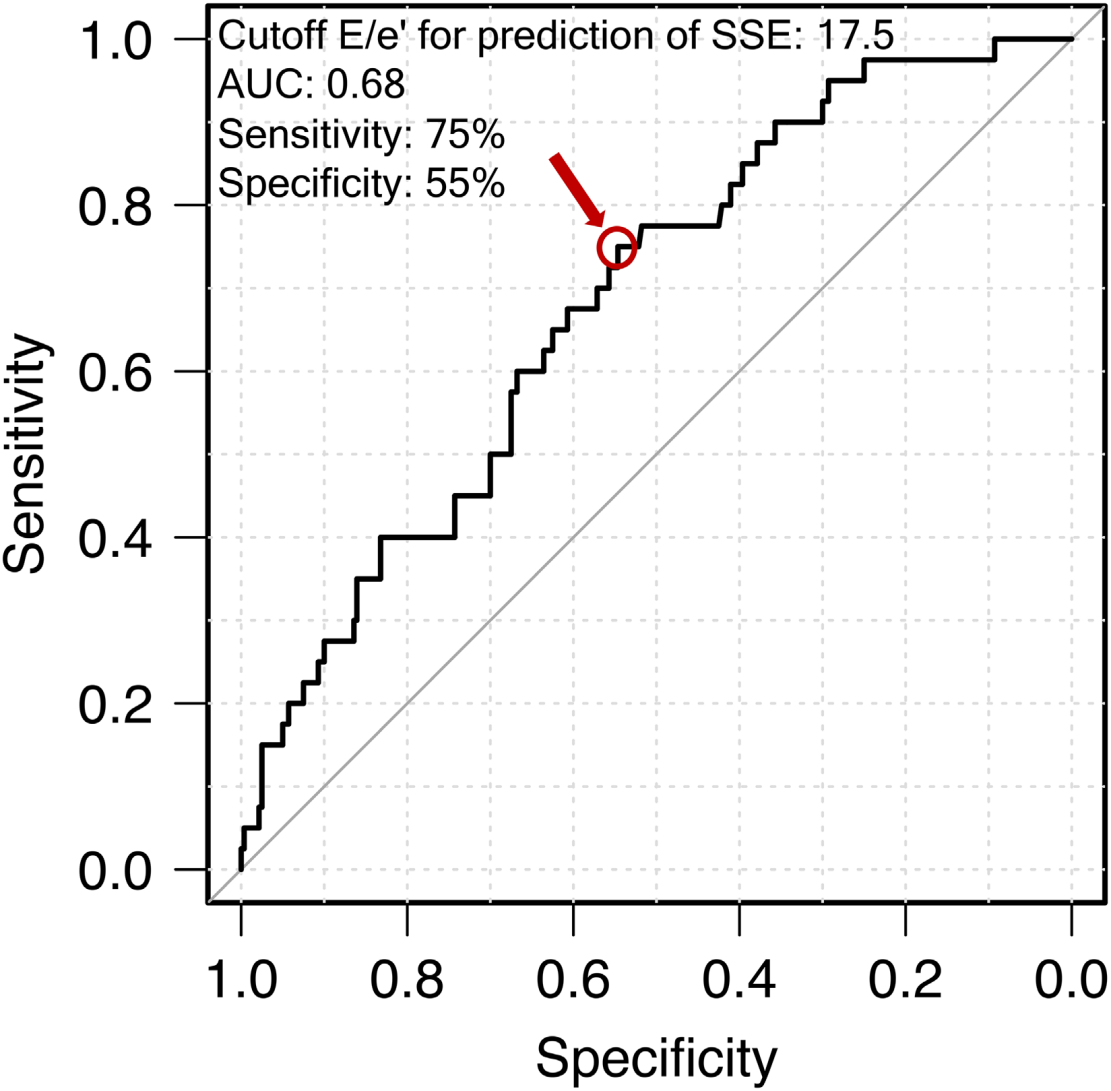
ROC curve showing the optimal E/e' cutoff value for prediction of SSE. The red circle shows that the optimal cutoff value is 17.5 (AUC 0.68, sensitivity 75%, specificity 55%). AUC, area under the curve; E, early diastolic peak flow velocity; e', early diastolic peak annular velocity; ROC, receiver‐operating characteristic; SSE, ischemic stroke or systemic embolism.

**FIGURE 2:**
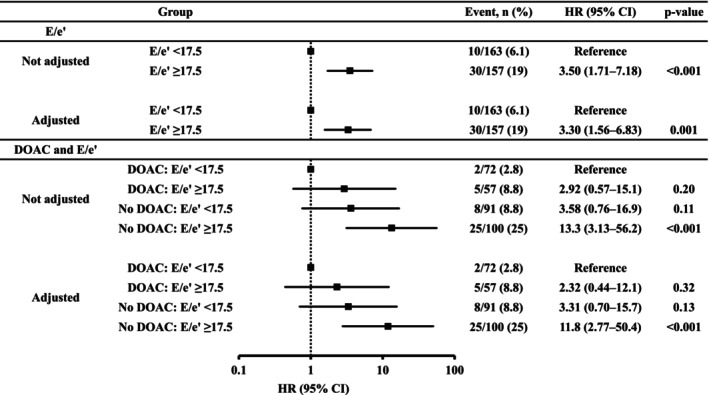
Results of univariate and multivariate Cox analyses of the impact of E/e' and DOAC use on the risk of SSE. Multivariate models for E/e' were adjusted for age, gender, and DOAC use. Multivariate models for E/e' and DOAC were adjusted for age and gender. CI, confidence interval; DOAC, direct oral anticoagulants; E, early diastolic peak flow velocity; e', early diastolic peak annular velocity; HR, hazard ratio; SSE, ischemic stroke or systemic embolism.

Table 3 shows the results of the Cox analysis of clinical characteristics predicting SSE. Heart rate and deceleration time were not included in the model because their proportional hazard values had not been established. In multivariate analysis with adjustment for age, gender, and factors that were significant in univariate analysis, age (HR 1.04, 95% CI 1.00–1.07, *p* = 0.034), DOAC use (HR 0.26, 95% CI 0.11–0.60, *p* = 0.002), and elevated E/e' (HR 1.05, 95% CI 1.02–1.08, *p* < 0.001) were significant independent predictors of SSE, whereas LVGLS and LARS were not. Analysis of the ROC curve revealed the appropriate cutoff value for E/e' to be 17.5 (area under the curve [AUC] 0.68, sensitivity 75%, specificity 55%; Figure 1). The adjusted multivariate Cox model showed the risk of SSE to be significantly higher in the high E/e' group (HR 3.30, 95% CI 1.56–6.83, *p* = 0.001; Figure 2) than in the low E/e' group (Table 3). After adjustment for age and gender and whether a DOAC was used, the risk of SSE was significantly higher in the high E/e' + no DOAC group (HR 11.8, 95% CI 2.77–50.4, *p* < 0.001; Figure 2) than in the low E/e' + DOAC group.

**TABLE 3:** Risk factors for SSE.

Risk factor	Univariate analysis			Multivariate analysis		
	HR	95% CI	*p*‐value	HR	95% CI	*p*‐value
Age	1.03	0.99–1.06	.054	**1.04**	**1.00–1.07**	**.034**
Female gender	**2.39**	**1.23–4.64**	**.010**	1.62	0.80–3.28	.18
Body mass index	0.98	0.92–1.04	.54			
Duration of AF (per 1 year)	1.01	0.95–1.08	.72			
History of smoking	0.65	0.34–1.25	.20			
eGFR	0.99	0.98–1.01	.47			
BNP (per 100 pg/mL)	1.02	0.97–1.06	.47			
Vascular disease	0.74	0.32–1.67	.46			
Hypertension	0.80	0.41–1.58	.53			
Diabetes	1.01	0.51–1.99	.98			
History of stroke	1.73	0.76–3.93	.19			
DOAC	**0.24**	**0.10–0.54**	**<.001**	**0.26**	**0.11–0.60**	**.002**
Warfarin	1.79	0.95–3.38	.071			
Antiplatelet agent	1.67	0.88–3.18	.12			
LVEF	1.01	0.98–1.03	.62			
LVMI	1.01	0.99–1.01	.087			
LAVI	1.00	0.99–1.01	.32			
E/e’ ratio	**1.06**	**1.03–1.09**	**<.001**	**1.05**	**1.02–1.08**	**<.001**
LVGLS	0.99	0.95–1.04	.76			
LARS	0.98	0.91–1.05	.53			

*Note*: Values in bold are statistically significant at *p* < .05.

Abbreviations: AF, atrial fibrillation; BNP, brain natriuretic peptide; CI, confidence interval; DOAC, direct oral anticoagulant; E, early diastolic peak flow velocity; e', early diastolic peak annular velocity; eGFR, estimated glomerular filtration rate; HR, hazard ratio; LARS, left atrial reservoir strain; LAVI, left atrial volume index; LVEF, left ventricular ejection fraction; LVGLS, left ventricular global longitudinal strain; LVMI, left ventricular mass index; SSE, ischemic stroke or systemic embolism.

### 3.3 Kaplan–Meier curves

**FIGURE 3:**
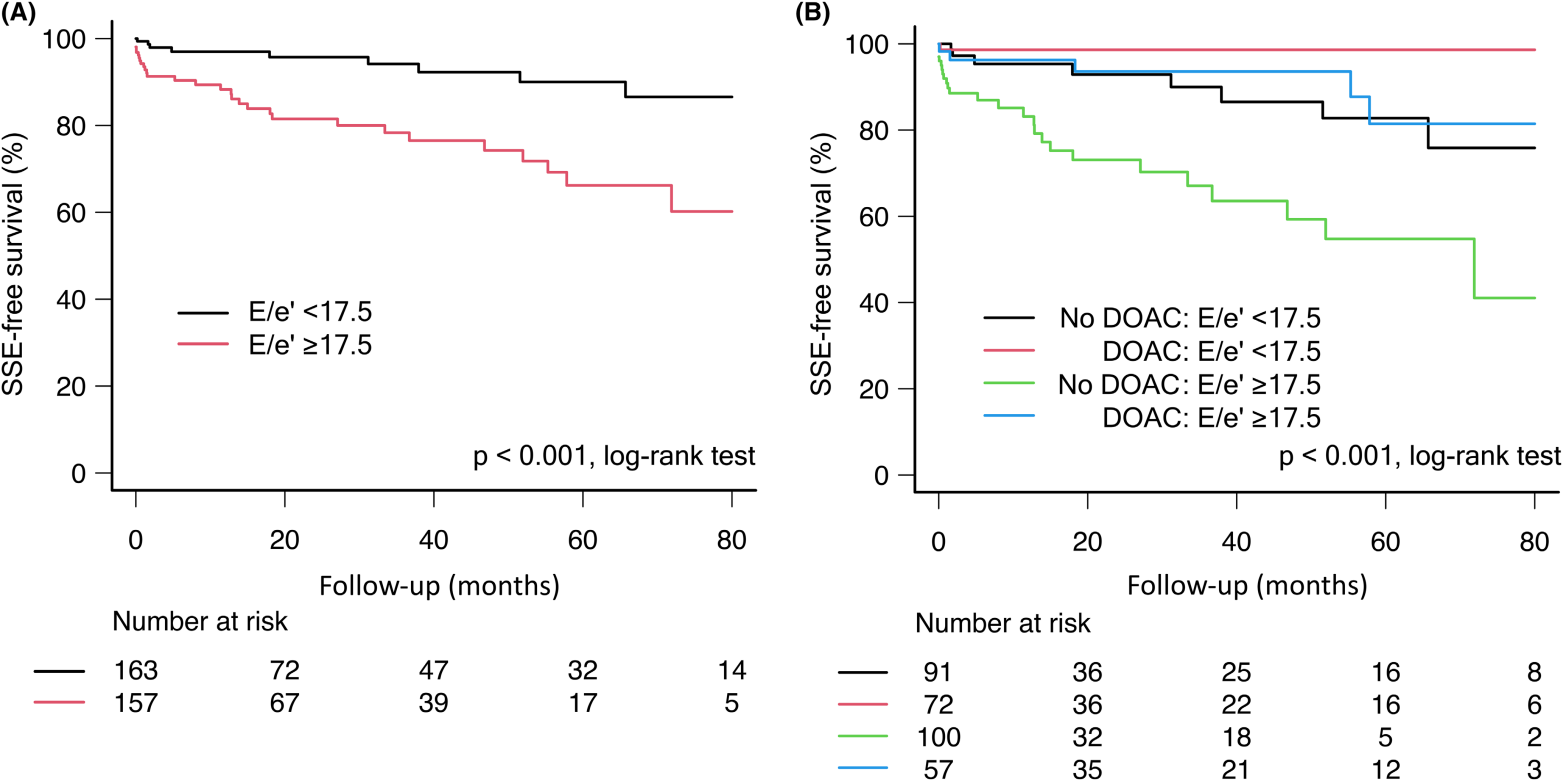
Kaplan–Meier SSE‐free survival curves. (A) Curve for E/e'. The black line indicates the low E/e' group and the red line indicates the high E/e' group. (B) Curves for E/e' and DOAC. The black line indicates the low E/e' + no DOAC group, the red line indicates the low E/e' + DOAC group, the green line indicates the high E/e' + no DOAC group, and the blue line indicates the high E/e' + DOAC group. DOAC, direct oral anticoagulants; E, early diastolic peak flow velocity; e', early diastolic peak annular velocity; SSE, ischemic stroke or systemic embolism.

The SSE‐free survival rate at 36 months was 86% (95% CI 80%–90%) in the overall patient population and 96% (95% CI 90%–99%) in the DOAC group. Figure 3(A) shows the Kaplan–Meier curves for the incidence of SSE in the event group and no‐event group when a cutoff E/e' value of 17.5 was used. After 36 months, the SSE‐free survival rate was 78% (95% CI 69%–85%) in the high E/e' group and 94% (95% CI 87%–97%) in the low E/e' group (*p* < .001, log‐rank test). Figure 3(B) shows the Kaplan–Meier curves for the incidence of SSE in the event and no‐event groups when divided further by DOAC use. After 36 months, the SSE‐free survival rate was 67% (95% CI 52%–78%) in the high E/e' + no DOAC group and 99% (95% CI 91%–100%) in the low E/e' + DOAC group (*p* < 0.001, log‐rank test).

### 3.4 Subgroup analyses

**FIGURE 4:**
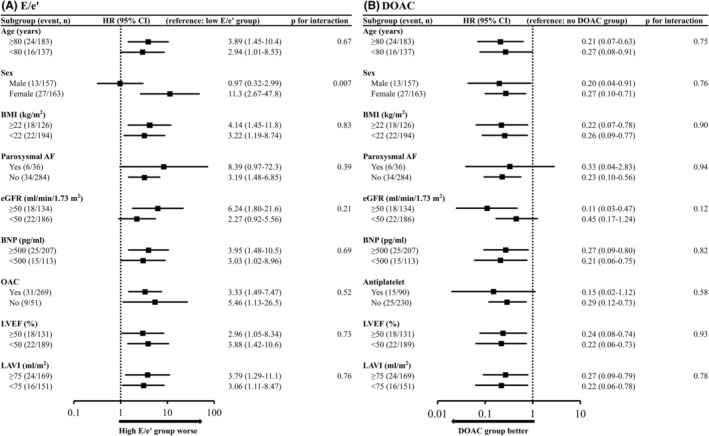
Subgroup analyses for E/e' and DOAC use. The HRs and 95% CIs for development of SSE according to E/e' (A) and DOAC use (B) are shown, along with *p*‐values for the interaction between each subgroup. AF, atrial fibrillation; BMI, body mass index; BNP, brain natriuretic peptide; CI, confidence interval; DOAC, direct oral anticoagulant; E, early diastolic peak flow velocity; e', early diastolic peak annular velocity; eGFR, estimated glomerular filtration rate; HR, hazard ratio; LAVI, left atrial volume index; LVEF, left ventricular ejection fraction; OAC, oral anticoagulant; SSE, ischemic stroke or systemic embolism.

The subgroup analyses of the development of SSE are shown for E/e' in Figure 4(A) and for DOAC use in Figure 4(B). There was a significant gender interaction for E/e', with E/e' having an increased ability to predict SSE in women. There was no statistically significant interaction for DOACs in any subgroup, but the efficacy of these agents was attenuated in patients with an estimated glomerular filtration rate of <50 mL/min/1.73 m^2^. No interaction was observed according to the use of antiplatelet agents.

### 3.5 Sensitivity analyses

Sensitivity for prediction of SSE was determined by adding the CHA₂DS₂–VASc score and whether antithrombotic agents were used in models 1 and 2 and adding echocardiographic values in models 3 and 4 (Table 4). In all models, the results were consistent with E/e' being an independent prognostic factor. An additional sensitivity analysis of 305 patients (SSE, *n* = 36) in whom echocardiography was performed after admission was consistent with the finding that E/e' was an independent predictor of SSE (Supplementary Table S1).

**TABLE 4:** Sensitivity analyses of risk factors for SSE events.

Risk factor	Model 1			Model 2		
	HR	95% CI	*p*‐value	HR	95% CI	*p*‐value
Age	1.03	0.99–1.06	.11	**1.03**	**1.00–1.05**	**.022**
CHA₂DS₂–VASc score	1.05	0.82–1.35	.70			
OAC	**0.24**	**0.10–0.57**	**.001**			
Warfarin				1.55	0.79–3.02	.20
Antiplatelet				1.51	0.79–2.88	.22
E/e' ratio	**1.07**	**1.04–1.11**	**<.001**	**1.06**	**1.03–1.09**	**<.001**

Risk factor	Model 3			Model 4		
	HR	95% CI	*p*‐value	HR	95% CI	*p*‐value
Age	**1.04**	**1.00–1.07**	**.026**	**1.04**	**1.00–1.07**	**.027**
LVEF	1.00	0.98–1.03	.81			
LAVI	1.00	0.99–1.00	.53			
E/e' ratio	**1.07**	**1.04–1.10**	**<.001**	**1.07**	**1.04–1.10**	**<.001**
LARS				1.02	0.95–1.11	.55
GLS				1.00	0.95–1.06	.91

*Note*: Values in bold are statistically significant at *p* < .05.

Abbreviation: BNP, brain natriuretic peptide; CI, confidence interval; E, early diastolic peak flow velocity; e', early diastolic peak annular velocity; eGFR, estimated glomerular filtration rate; GLS, global longitudinal strain; HR, hazard ratio; LARS, left atrial reservoir strain; LAVI, left atrial volume index; LVEF, left ventricular ejection fraction; OAC, oral anticoagulants; SSE, ischemic stroke or systemic embolism.

## 4 DISCUSSION

In this study, we investigated the association of E/e' and DOAC use with the development of SSE in patients with AF and HF. The SSE‐free survival rate at the 36‐month follow‐up was 86%, which is lower than the rate found in a previously reported cohort study performed during the same time period.^4^ This difference may be attributed to the fact that the patients with HF in the present study were older, and therefore more severely ill than those in the previous study, and a greater proportion of our patients were on warfarin instead of DOAC. In multivariate analysis, older age, no DOAC therapy, and elevated E/e' were independent predictors of SSE, and these results were generally consistent with those of the sensitivity and subgroup analyses. To the best of our knowledge, this is the first study in Japan to identify E/e' as a predictor of SSE in patients with acute HF complicated by AF.

### 4.1 Relationship between atrial cardiopathy, OAC use, and stroke

Stroke caused by AF has been thought to be a consequence of the formation of thrombus in the LA appendage as a result of blood pooling caused by a lack of organized atrial contraction. However, a condition known as “atrial cardiopathy,” in which thrombosis occurs in the left atrium without being associated with arrhythmia, has recently been identified.^16^ Atrial cardiopathy reflects atrial remodeling caused by the combined effects of various atherosclerotic diseases such as hypertension, diabetes mellitus, and dyslipidemia and is thought to cause stroke by the formation of a thrombosed atrial matrix.^17^ There have been reports of atrial cardiopathy being associated with an increased risk of stroke even in the absence of AF or a history of stroke.^17^ The combination of AF and HF synergistically increases fibrosis in the left atrium and exacerbates atrial cardiopathy.^18^ However, the true risk of stroke is difficult to determine because it has been indirectly assessed by the CHADS_2_ score, which is used to determine the indication for anticoagulation, without assessment of the severity of atrial cardiopathy. A further problem is that atrial cardiopathy has not yet been defined, so it is necessary to diagnose the disease using multiple modalities, including electrocardiography, echocardiography, cardiac ultrasound, cardiac magnetic resonance imaging, and the brain natriuretic peptide level. There is also a need for a guideline that can be used to evaluate the severity of the disease.

The rate of OAC use in our study was 84%, which is low by current medical standards considering the severity of the disease. However, in the previously reported cohort study from the same period, OAC use in the patient group with the same CHADS_2_ score as that in the present study was around 70%.^4^ Therefore, the rate was better in our study. The prevalence of DOAC use in Japan at that time was also low at around 50%,^4^ and the same trend was observed in our study. The evidence for the use of DOACs was weaker than that in the current guidelines,^2^ which may have contributed to the low rate of DOAC and even OAC use. Furthermore, the median HAS‐BLED score in our patients was around 2–3, which suggests a high risk of bleeding, and it is possible that patients at high risk of SSE were considered to be at increased risk of bleeding.

A prospective observational study found that elderly patients with AF on DOACs had fewer stroke and major bleeding events than their counterparts on warfarin, regardless of the presence or absence of HF.^19^ In the present study, DOAC use did not show a significant interaction between the subgroups but reduced renal function attenuated the benefit of using DOACs. It is possible that patients with reduced renal function were prescribed DOACs at an inappropriate dose, were switched to warfarin, or discontinued anticoagulant therapy. Nevertheless, DOACs have been reported to be effective and safe even in patients with renal failure.^20^ Therefore, clinicians can prescribe these agents at appropriate doses without concern about bleeding. Our main analysis showed that the risk of stroke was 12 times higher in DOAC‐naive patients with an increased E/e' than in the DOAC‐treated patients with a decreased E/e', suggesting that careful follow‐up is needed when discontinuing DOAC therapy in patients with increased E/e'. These findings indicate that DOACs should be the first choice of anticoagulation therapy in patients with HF complicated by AF and the stroke risk should be reevaluated based on indices such as E/e' when anticoagulation therapy is discontinued because of the risk of bleeding.

### 4.2 Echocardiographic predictors of stroke in patients with AF

In previous reports, the main predictors of stroke on echocardiography in patients with AF have been LVEF, LA diameter, LA volume index, LA appendage flow, E/e', LVGLS, and LARS.^21^, ^22^, ^23^, ^24^, ^25^, ^26^, ^27^, ^28^, ^29^ In our sensitivity analysis adjusted for these predictors, only E/e' was an independent predictor of stroke. Patients who have HF with preserved LVEF have been reported to have a higher risk of stroke than those who have HF with reduced LVEF.^21^ Furthermore, an increased LA volume index indicates structural remodeling as a result of chronically elevated LV end‐diastolic pressure, which is associated with an increased risk of stroke.^24^, ^27^ E/e' starts to rise as LA pressure increases in addition to structural remodeling because of the inclusion of not only tissue Doppler but also the inflow of blood in the left ventricle in the evaluation.^30^ Therefore, an increase in E/e' is significantly correlated with an increase in LV end‐diastolic pressure at the onset of acute HF^8^ and has been reported to be a predictor of stroke in patients with AF because of the increased risk of stroke owing to worsening HF.^21^, ^23^ In our subgroup analysis, the ability of E/e' to predict SSE was better in women, which is in line with the findings of a previous cross‐sectional study.^31^ Given the prevalence of older patients with HF with preserved ejection fraction among women, LA dysfunction may be more pronounced in women when stroke risk increases equally between the genders.

LARS obtained by the two‐dimensional speckle tracking method has likewise been reported to be a predictor of stroke in patients with AF.^22^, ^25^ LARS has been described as an excellent indicator when screening for LA dysfunction because it starts to decline early in the functional remodeling that occurs in patients who have not yet developed LA enlargement.^30^ However, it has been pointed out that LARS may not differentiate between patients or affect the prognosis considering that all patients with AF and HF are severely ill and have advanced structural remodeling.^32^ Again, in the present study, structural remodeling was markedly advanced with a mean LA volume index of 90 mL/m^2^ and the mean LARS was low at 9.6%, with no significant results regarding the development of SSE. This suggests that LARS may be a good predictor of stroke in patients in the early stages of HF, when LA dysfunction has only recently started, and E/e' is a better predictor in patients with advanced HF.

### 4.3 Stroke prevention in patients with AF and HF

In this study, adjustment for the CHADS_2_ score did not attenuate the predictive ability of E/e'. Therefore, even in patients with AF and HF who have a low CHADS_2_ score, an elevated E/e' would be expected to indicate a high risk of stroke. Indeed, a small number of strokes have been reported even in patients with a low CHADS_2_ score.^33^, ^34^ The guidelines recommend continued anticoagulation if the CHADS_2_ score is >2 after catheter ablation,^35^ but it may be advisable to continue anticoagulation even in patients with no improvement in E/e'. Our present findings also suggest that the LA appendage should be routinely evaluated for thrombus before initiation of rhythm control in elderly patients with an elevated E/e' and not on a DOAC (including those on warfarin).

There has been a report of a possible association between elevated E/e' and development of atherothrombotic stroke in a group of patients without a history of AF.^36^ Given that progression of atherosclerosis is a risk factor for atherothrombosis, it is possible that the present study included some patients in whom anticoagulation alone was inadequate. Furthermore, it has been reported that the rate of recurrent ischemic stroke is higher in patients with AF who develop ischemic stroke despite anticoagulation therapy than in those who do not receive anticoagulation.^37^ This finding implicates pathogenic mechanisms other than AF‐related stroke, and patients with a history of stroke may need to be monitored for adherence with medication and screened for arterial stiffness by echocardiography of the internal carotid artery and magnetic resonance angiography of the head. Although there is controversy regarding the efficacy and safety of combined anticoagulant and antiplatelet therapy in this patient population, in a cohort study of patients with AF, combination therapy was associated with an increased risk of major bleeding and stroke, regardless of whether atherosclerotic disease was present.^38^ Similar results were reported for patients with AF and a history of ischemic stroke,^39^ and there is currently much negative evidence regarding combination therapy.

It is likely that some patients with more severe atrial cardiopathy remain at high risk of stroke despite anticoagulation therapy. Catheter ablation for rhythm control and anticoagulation with addition of LA appendage closure to reduce the risk of stroke have also been reported to be effective.^40^, ^41^ Considering that rhythm control is difficult to achieve in some patient groups owing to the progression of HF stage, there is a need for agents that can slow the progression of atrial cardiopathy. Randomized controlled trials are needed to determine whether new agents for HF such as angiotensin receptor/neprilysin inhibitors and sodium–glucose cotransporter 2 inhibitors, which have been reported to improve LA dysfunction by reverse remodeling,^42^, ^43^ can reduce the stroke risk in this patient population. Given that other diseases, including lacunar infarction, branch atheromatous disease, and cryptogenic stroke (paradoxical embolism, vasculitis, malignancy) are also possible causes,^44^ clinicians will be required to evaluate the mechanism of stroke in each patient.

### 4.4 Limitations

This study has several limitations. First, it had a single‐center, retrospective design and included a small study population. Furthermore, the number of pulmonary vein isolation procedures was low because the hospital did not become a catheter ablation facility until 2018. Patients who were referred to other hospitals for ablation and not subsequently referred back to our hospital were lost to follow‐up, which presumably contributed to the low ablation rate in patients who were available for follow‐up. The differences between our results and those of related studies probably reflect differences in study populations, measurement environments, and the treatment interventions used. Second, surveillance bias and detection bias could not be completely eliminated. In general, SSE is characterized by a severe clinical course, making surveillance bias unlikely. On the other hand, the incidence of SSE was higher in our patients than that reported for a general HF cohort,^4^ and more patients dropped out early. Patients at high risk of stroke may have received preferential follow‐up, which could have resulted in detection bias that increased the incidence of SSE. Third, echocardiograms were performed within 30 days before or after the date of admission, and some cases may not reflect values from the acute phase of HF. However, the median time from admission to echocardiography in this study was only 6 days (IQR 3–9 days) and the results were consistent in the sensitivity analysis with patients in whom echocardiography was performed after admission. There is also the impact of accuracy and reproducibility in echocardiography, which we have previously reported in other analyses.^13^ Finally, we cannot rule out the possibility of unmeasured confounding factors.

## 5 CONCLUSION

We have found a significant association of E/e' and no DOAC use with the development of SSE in patients hospitalized for acute HF with AF. This finding suggests that DOACs should be the first choice for patients with elevated E/e' and that there is a need for aggressive additional prophylaxis and careful follow‐up. LARS can detect LA dysfunction at an earlier stage, but its predictive ability may be reduced in patients with advanced HF. E/e' reflects the severity of LA remodeling and may be useful for identifying patients with atrial cardiopathy who have more advanced LA fibrosis and an increased risk of stroke.

## FUNDING INFORMATION

This research received no specific grant from any funding agency in the public, commercial, or not‐for‐profit sectors.

## CONFLICT OF INTEREST STATEMENT

The authors declare that there are no conflicts of interest.

## ETHICS STATEMENT

The study was approved by the institutional review board of the National Center for Global Health and Medicine (NCGM‐S‐004777‐00).

## PATIENT CONSENT STATEMENT

The requirement for written informed consent was waived by the institutional review board. However, patients were given the opportunity to decline participation via the opt‐out route on the hospital website.

## PERMISSION TO REPRODUCE MATERIAL FROM OTHER SOURCES

Not applicable.

## CLINICAL TRIAL REGISTRATION

Not applicable.

## Supporting information


Table S1.

## Data Availability

The data underlying this article cannot be shared publicly without compromising the privacy of the study participants but are available from the corresponding author upon reasonable request.
