# Supplementary material for: Impact of left ventricular diastolic function and direct oral anticoagulant use for predicting embolic events in patients with heart failure and atrial fibrillation
Source: J Arrhythm. 2024 Mar 26;40(3):489–500. doi: 10.1002/joa3.13031 (PMC11199849; doi:10.1002/joa3.13031)
Supplement: Supplementary file 1 — Table S1. [file JOA3-40-489-s001.docx]

Supplementary Table 1. Sensitivity analyses for SSE events in patients in whom echocardiography was performed after hospitalization

|  | Univariate analysis | | | Multivariate analysis | | |
| --- | --- | --- | --- | --- | --- | --- |
| Risk factor | HR | 95% CI | p-value | HR | 95% CI | p-value |
| Age | **1.04** | **1.00-1.07** | **0.046** | 1.03 | 0.99-1.07 | 0.13 |
| Female sex | **2.19** | **1.09–4.42** | **0.028** | 1.65 | 0.78–3.47 | 0.19 |
| Body mass index | 0.97 | 0.90–1.04 | 0.37 |  |  |  |
| Duration of AF (per 1 year) | 1.02 | 0.95–1.09 | 0.66 |  |  |  |
| History of smoking | 0.71 | 0.36–1.42 | 0.34 |  |  |  |
| eGFR | 0.99 | 0.98–1.02 | 0.87 |  |  |  |
| BNP (per 100 pg/ml) | 1.02 | 0.97–1.06 | 0.49 |  |  |  |
| Vascular disease | 0.84 | 0.37–1.93 | 0.69 |  |  |  |
| Hypertension | 0.78 | 0.38–1.60 | 0.50 |  |  |  |
| Diabetes | 0.96 | 0.46–2.01 | 0.92 |  |  |  |
| History of stroke | 1.80 | 0.74–4.38 | 0.19 |  |  |  |
| DOAC | **0.27** | **0.12–0.62** | **0.002** | **0.29** | **0.12–0.66** | **0.004** |
| Warfarin | 1.70 | 0.87–3.33 | 0.12 |  |  |  |
| Antiplatelet agent | 1.63 | 0.82–3.24 | 0.16 |  |  |  |
| LVEF | 1.00 | 0.98–1.03 | 0.81 |  |  |  |
| LVMI | 1.01 | 0.99–1.01 | 0.21 |  |  |  |
| LAVI | 1.00 | 0.99–1.01 | 0.76 |  |  |  |
| E/e' ratio | **1.06** | **1.03–1.10** | **<0.001** | **1.05** | **1.02–1.09** | **0.002** |
| LVGLS | 1.00 | 0.95–1.06 | 0.89 |  |  |  |
| LARS | 0.97 | 0.90–1.05 | 0.50 |  |  |  |

Values in bold are statistically significant at p < 0.05. AF, atrial fibrillation; BNP, brain natriuretic peptide; CI, confidence interval; E, early diastolic peak flow velocity; e', early diastolic peak annular velocity; eGFR, estimated glomerular filtration rate; HR, hazard ratio; LARS, left atrial reservoir strain; LAVI, left atrial volume index; LVEF, left ventricular ejection fraction; LVGLS, left ventricular global longitudinal strain; LVMI, left ventricular mass index; DOAC, direct oral anticoagulant; SSE, ischemic stroke or systemic embolism
